# Do Concomitant Systematic Biopsies Add to Fusion Targeted Biopsies in the Diagnosis and Management of Clinically Significant Prostate Cancer?

**DOI:** 10.5152/tud.2023.22221

**Published:** 2023-05-01

**Authors:** Alice Thompson, Venkat Eguru, Sohail Moosa, Yeung Ng

**Affiliations:** 1 University Hospital of Wales Healthcare NHS Trust, Northern Ireland, UK; 2 Hywel Dda University Health Board, Northern Ireland, UK

**Keywords:** Prostate cancer, systematic biopsy, targeted biopsy

## Abstract

**Objective:**

Magnetic resonance imaging targeted biopsy clearly detects more clinically significant prostate cancer than systematic biopsy. Whether concomitant systematic biopsy adds to targeted biopsy in the detection of clinically significant prostate cancer remains uncertain. The primary outcome measure of this study was to ascertain the percentage of clinically significant prostate cancer on systematic biopsy missed by targeted biopsy. Furthermore, we sought to determine whether systematic biopsy results influenced the clinical management of patients.

**Materials and Methods:**

This prospective observational study included all men undergoing Fusion targeted biopsy in our Health Board. All men had PI-RADS scores of 3-5 on magnetic resonance imaging. Histology from targeted biopsy and systematic biopsy was reviewed to determine any additional benefit of performing systematic biopsy. Clinical outcomes were also reviewed. Clinically significant prostate cancer was defined by (i) International Society of Urological Pathology ≥ 2 and (ii) UCL criteria of any primary Gleason 4 or core length ≥ 6 mm.

**Results:**

A total of 104 men were included in the study of whom 18 patients were biopsy naïve, 65 had at least 1 previous negative biopsy, and 20 had previous biopsies that showed clinically insignificant cancer. The percentage of clinically significant prostate cancer missed on targeted biopsy was between 9.1% and 11.1%. Moreover, 17.1% of patients with clinically significant prostate cancer would not have proceeded to radical treatment if the systematic biopsy had not been performed.

**Conclusion:**

Our data support a growing field of evidence that although magnetic resonance imaging targeted biopsy is more sensitive than systematic biopsy at detecting clinically significant prostate cancer, systematic biopsy adds to the number of patients diagnosed with clinically significant prostate cancer in those already undergoing prostate biopsy.

**Main Points**

- Minimising false negative prostate biopsies is fundamental for management.
- About 10% of patients with negative targeted biopsies had clinically significant cancer on concomitant systematic biopsies.
- This impacts clinical decision-making.
- Systematic biopsy should be performed at the same time as targeted biopsy.

## Introduction

Pre-biopsy multiparametric prostate MRI (mpMRI) is the new paradigm, due to extensive evidence from PROMIS,^1^ PRECISION,^2^ and MRI First trials,^3^ which is reflected in its recommendation in EAU^4^ and NICE^5^ Guidelines. These trials confirm that MRI and MRI targeted biopsy (TB) detect more clinically significant prostate cancer (csPCa) than systematic biopsy (SB), yet the same evidence shows that mpMRI misses 11%-28% of csPCa.^1^ Whether concomitant SB should be performed at the same time remains controversial.^6–8^

Radical treatment of prostate cancer is associated with significant side effects.^9,10^ The difference in malignant progression and mortality rates associated with the different histological grades of prostate cancer is significant. Studies with follow-up over 15-29 years show low rates of prostate cancer-related deaths in those with Gleason 6 prostate cancer in watchful waiting (3%)^11^ or active surveillance (AS) groups (1.5%),^12^ whereas radical prostatectomy (RP) specimen analysis reveals that higher Gleason grade or International Society of Urological Pathology (ISUP) grade^13^ predicts earlier biochemical progression, clinical progression, and worse cancer-specific survival.^14,15^

This has resulted in clinicians aiming to avoid overdiagnosis of low-risk prostate cancer due to biopsy risks,^16^ while attempting to not miss csPCa.

This endeavor has led to targeting lesions seen on biparametric MRI or mpMRI of prostates. Despite this, we know that even TB of lesions misses csPCa,^1^ and inaccuracies are discovered with both upgrading and, to a lesser extent, downgrading of final histological diagnoses when RP specimen histology has been reviewed.^17–19^

Overtreatment remains a risk in biopsy-diagnosed prostate cancer. This is suggested by UK RP data showing that 39.6% of men who had RP between 2011 and 2016 had low-risk ISUP 1 disease on RP histology specimens.^19^ Confidence in an accurate biopsy could reduce this overtreatment, by allowing clinicians and patients to better judge the likelihood of progression and mortality and be more informed to decide whether they proceed with a treatment that reduces their quality of life.^11,12^

The definition of csPCa varies. PRECISION trial criteria for csPCa is ISUP ≥ 2 (Gleason ≥ 3 + 4).^2^ The PROMIS trial used the UCL 1 criteria of any primary Gleason 4 or above, or core length ≥ 6 mm.^1^ Classification by grade simplifies statistical analysis, but patient factors and tumor factors will impact individual clinical significance, with age, Gleason score, tumor volume, surgical margin, and transitional zone tumors having a significant impact on survival outcomes.^17,20^

The PROMIS trial showed mpMRI detects more csPCa than SB and detects less insignificant cancer which can avoid the need for biopsy in 27% of men with PI-RADS (Prostate Imaging Reporting and Data System) 1-2 lesions.^1^ The PRECISION trial showed that biopsying PI-RADs 3-5 lesions only with mpMRI-influenced TB detects more csPCa than blind SB in patients with PI-RADS 1-5 lesions. Furthermore, TB detects less clinically insignificant cancers and 28% of men with PI-RADS 1-2 lesions on mpMRI avoided a biopsy.^2^

However, although these and other studies^21^ suggest mpMRI and mpMRI TB have a better clinically significant cancer detection rate (csCDR) than SB, this does not mean that TB detects all csPCa, which leads to the question; should TB be performed in isolation? Or does concomitant SB add to the diagnosis of csPCa.

Here, we present our center’s data on the additional detection of csPCa found on MRI TB and SB. This study sought to determine whether concomitant SB taken in conjunction with TB using Fusion software increased the csPCa detection rate and clinical outcomes of all men undergoing Fusion TB in our Trust.

## Materials and Methods

### Study Design

We conducted this study as a prospective observational study. It included all men undergoing Ultrasound-MRI Fusion TB in our Healthboard, with the aim of evaluating SB and TB histology results and their impact on clinical outcomes.

The primary outcome measure was to ascertain the percentage of csPCa detected on SB missed by TB. Furthermore, we sought to determine whether SB results influenced the clinical management of patients.

The csPCa was defined using 2 criteria: (i) ISUP ≥ 2 (Gleason ≥ 3 + 4); and (ii) UCL 1 criteria used in the PROMIS trial^1^ (i.e., any primary Gleason 4 or core length ≥ 6 mm). The definition of csPCa varies and therefore both definitions were used in order to satisfy this ambiguity seen in daily clinical practice.

Ethics committee approval was not required for this observational study. This project was registered as a quality improvement project within the Health Board. We referred to the Research Authority Decision Tool and this project does not constitute research requiring REC/HRA approval.

### Imaging and Biopsy Technique

All men had undergone biparametric MRI using a Siemens Aera 1.5 Tesla MRI scanner with T2-weighted imaging and diffusion-weighted imaging (DWI) sequence acquisition, with selected patients undergoing multiparametric 1.5 Tesla MRI using gadolinium enhancement.

All cases had been selected to undergo Fusion TB by a dedicated MDT meeting with a senior uro-radiologist and 2 experienced consultant urologists with all MRI scores of between 3 and 5 based on the 5-point PI-RADS v2 scale.^22^

The decision to undergo Fusion TB over cognitive TB was principally based on MRI findings inconsistent with previous negative or clinically insignificant biopsy results or lesions on MRI pedicted to be difficult to target using cognitive TB. For example; lesionstypically less than 8 mm in diameter and/or in larger prostate gland volumes, and/or in areas thought to be less amenable to non-fusion targeting, typically anteriorly placed or in the prostate base.

Fusion TB was carried out under general anesthetic and was performed using the Hitachi Preirus system with RVS fusion platform using an external magnetic field generator tracking mechanism. Biopsies were taken using Hitachi biplanar probe via a trans-rectal approach or with a Hitachi Noblus US system using the BIOPSEE fusion platform via a trans-perineal approach. All men were provided written consent and were given pre-operative 500 mg ciprofloxacin and 160 mg intramuscular gentamicin plus post-procedural 1 g per rectal metronidazole and then completed a 3-day course of ciprofloxacin 500 mg twice a day as antibiotic prophylaxis.

The SBs were taken concomitantly as 12 core peripheral zone biopsies either trans-rectally or trans-perineally depending on the mode of fusion biopsy. If SB had been taken within 6 months prior to FB, then the histology results from those samples were used for the analysis.

Histological results, MRI reports and images, and clinical outcomes were retrospectively reviewed using the Clinical Portal.

## Results

A total of 104 men were included in the study of whom 18 patients were biopsy naïve, 65 had at least 1 previous negative biopsy, and 20 had previous biopsies that showed clinically insignificant cancer. Seventy-one had concomitant 12 core trans-rectal SB with the remaining patients having had SB within the preceding 6 months.

All patients had MRI lesions that had been given a PI-RADS v2 score of 3-5. These were located in the anterior portion of the prostate in 51/104 patients (49%), 15/104 had apex lesions (14%), and 8/104 were located in the base of the prostate (7.7%).

The characteristics of the men included are detailed in Table 1. The 104 men included in the study were aged between 52 and 82 with a median age of 68. The median Prostate Specific Antigen (PSA) was 9.3 (range 1.3-35.5) and prostate volume range was 12-227 cc (median 60 cc).

**Figure 1.**
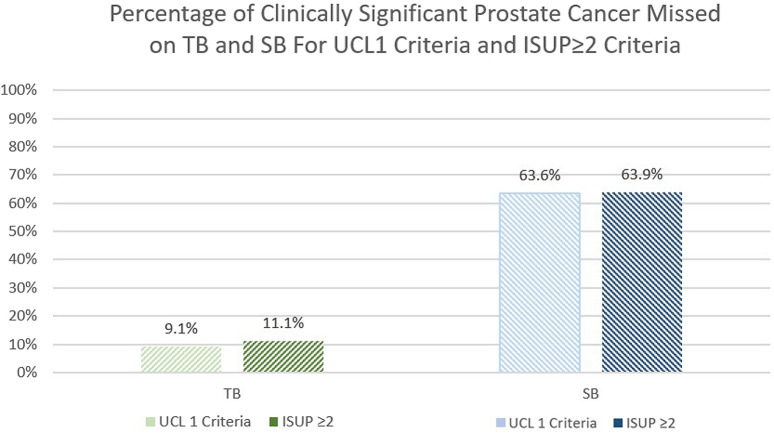
Percentage of csPCa missed on TB and on SB as defined by UCL1 criteria: (any primary Gleason 4 or core length ≥ 6 mm) and ISUP ≥ 2 criteria: (Gleason ≥ 3 + 4). csPCa, clinically significant prostate cancer; SB, systematic biopsy; TB, targeted biopsy.

The percentage of csPCa missed on TB was between 2/22 (9.1%) (ISUP≥/Gleason ≥ 3 + 4) and 11.1% (4/36) (UCL 1 criteria) (Figure 1).

**Figure 2.**
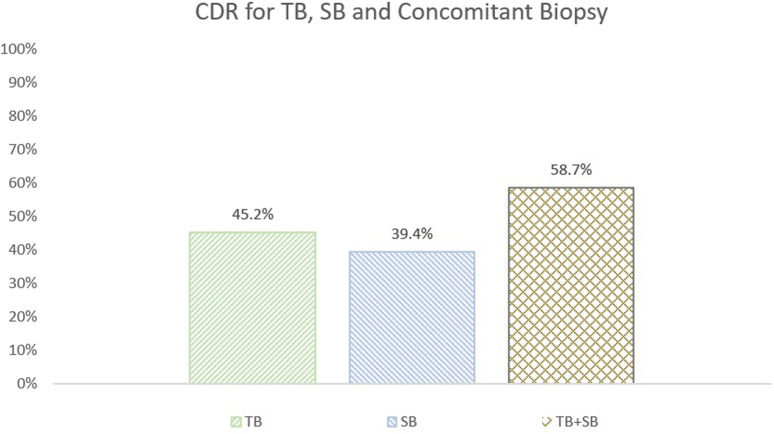
Cancer detection rate (CDR) for TB, SB, and concomitant biopsy. SB, systematic biopsy; TB, targeted biopsy.

**Figure 3.**
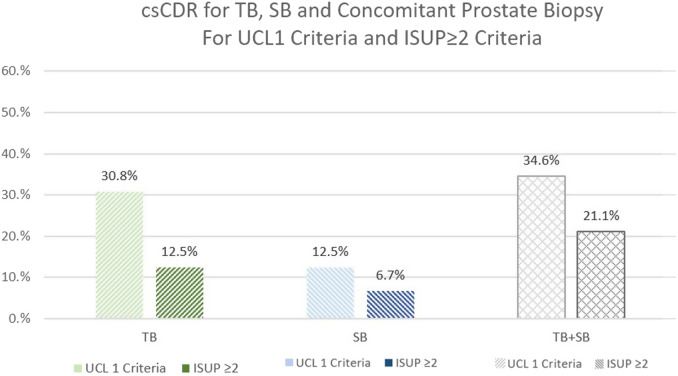
Clinically significant cancer detection rate for TB, SB, and concomitant biopsy. As defined by 2 different criteria of csPCa: UCL1 criteria: (any primary Gleason 4 or core length ≥ 6 mm) and ISUP ≥ 2: (Gleason ≥ 3 + 4). csPCa, clinically significant prostate cancer; SB, systematic biopsy; TB, targeted biopsy.

Prostate cancer was found in 58.7% (61/104) of the participants with the combined results from TB and SB. The TB (alone) CDR was 47/104 (45.2%), and the SB (alone) CDR was 41/104 (39.4%) (Figure 2). The csPCa was detected in between 22/104 (21.2%) and 36/104 (34.6%) (any ISUP ≥ 2 and UCL 1 criteria, respectively) (Figure 3).

**Figure 4.**
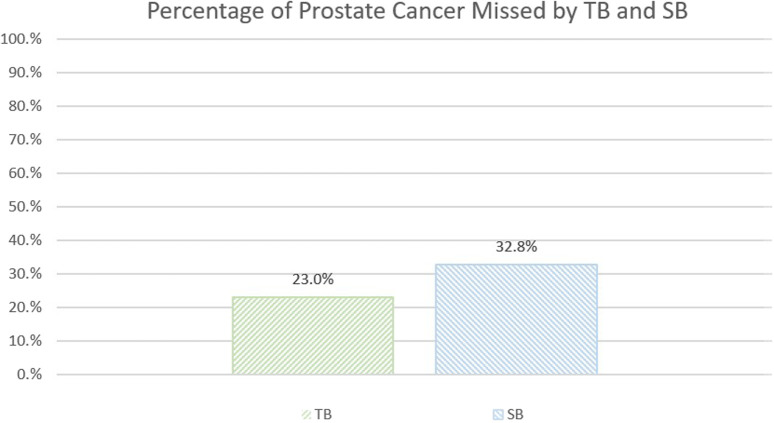
Prostate cancer missed by TB and SB as a percentage of those diagnosed with PCa by a combined technique. PCa, prostate cancer; SB, systematic biopsy; TB, targeted biopsy.

The TB alone missed 23% (14/61) of all PCa, and SB alone missed 32.8% (20/54) of all PCa (Figure 4). The SB missed between 63.6% (14/22) (ISUP ≥ 2) and 63.9% (23/36) (UCL 1 criteria) of csPCa. (Figure 1).

**Figure 5.**
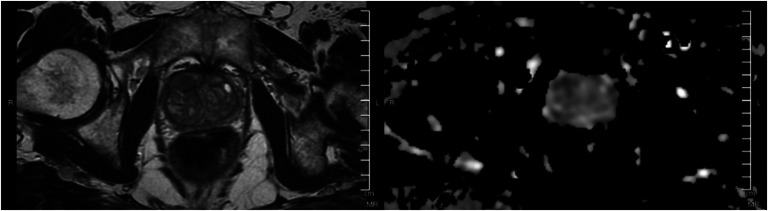
MRI images used for Fusion biopsy. T2W images (left) and ADC images (right) show right anterior lesion. All SBs were negative. 3/3 TBs were positive for Gleason 4 + 3 PCa. This patient went on to have radical radiotherapy. MRI, magnetic resonance imaging; PCa, prostate cancer; SB, systematic biopsy; TB, targeted biopsy.

**Figure 6.**
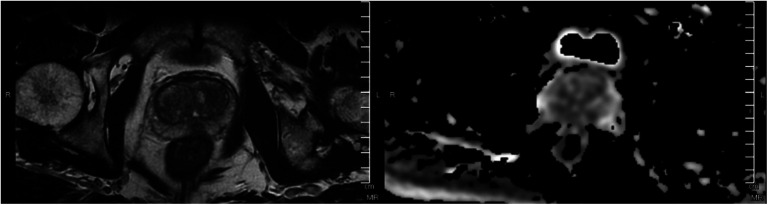
MRI images used for Fusion biopsy. T2W (T-2 weighted imaging) images and ADC (Apparent diffusion coefficient) images show a left paramedian peripheral zone lesion. Eleven systematic biopsies were negative. A total of 1/5 targeted biopsies showed 4 + 4 PCa. He went to have a radical prostatectomy which showed Gleason 4 + 3 T3a N0M0 disease. MRI, magnetic resonance imaging; PCa, prostate cancer.

This is far in excess of those missed by TB, but this is expected and is why TBs are recommended in guidelines. Figures 5 and 6 show examples of patients where only the TB showed csPCa.

**Figure 7.**
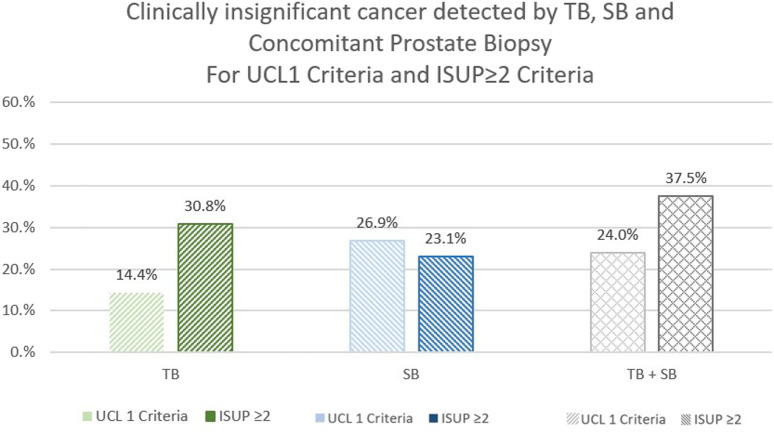
Clinically insignificant cancer detected by TB, SB, and concomitant biopsy. Comparison of percentage of UCL1 criteria: (any primary Gleason 4 or core length ≥ 6 mm) and ISUP ≥ 2: (Gleason ≥ 3 + 4). SB, systematic biopsy; TB, targeted biopsy.

Clinically insignificant cancers were detected by both techniques, with TB outperforming SB at detecting fewer clinically insignificant cancers. The TB detected between (15/104) 14.4% and (32/104) 30.8% of clinically insignificant cancers for UCL 1 criteria and ISUP ≥ 2, respectively. The SB detected between (28/104) 26.9% and (24/104) 23.1% of clinically insignificant cancers according to which histology criteria for clinical significance was used; defined by either UCL 1 criteria or ISUP ≥ 2, respectively. Combined biopsy detected clinically insignificant cancer in (25/104) 24% and (39/104) 37.5% of men (<UCL1 criteria and ISUP 1, respectively) (Figure 7).

**Figure 8.**
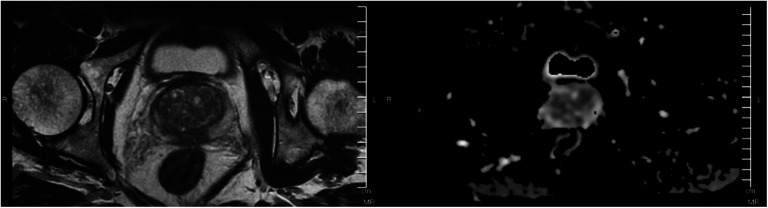
The MRI images of patient 1 (in Table 2). The T2W (T2-weighted) images and ADC (Apparent diffusion coefficient) images show bilateral paramedian lesions. All target biopsies were negative. The SB from the left showed 1/5 Gleason 4 + 4. This patient had a radical prostatectomy which showed multifocal Gleason 3 + 4. SB, systematic biopsy; MRI, magnetic resonance imaging.

**Figure 9.**
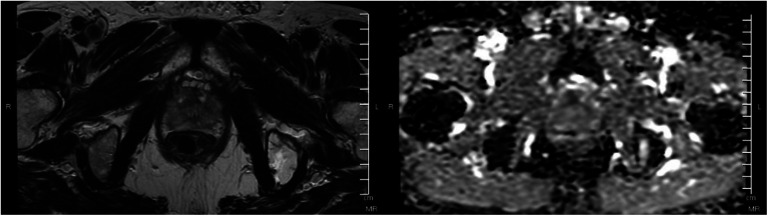
The MRI images of patient 2 (in Table 2). The T2W (T2-weighted) images and ADC (Apparent diffusion coefficient) images show the right apex anterior lesion. Targeted biopsy would have given this patient a category of clinically significant prostate cancer according to UCL1 criteria only. The SB resulted in a Gleason 4 + 3 biopsy which is the more influential factor leading to treatment. This patient had radical radiotherapy. SB, systematic biopsy; TB, targeted biopsy.

In this cohort, 35 men were offered radical treatment on the basis of their combined biopsy results. Case analysis showed that of the 35 patients offered radical treatment, 29 patients could have had their treatment decisions based on TB alone. In the remaining 6 patients (Table 2), TB was either benign (1) or clinically insignificant (4) and the sixth patient was significant on the UCL 1 criteria but the presence of ISUP ≥ 2 histology on the SB was the more influential factor in proceeding with radical treatment. This patient could have been offered AS or radical treatment based on TB results, but the SB found unequivocally csPCa, which would have made AS inappropriate. MRI images for 2 of these patients can be seen in Figures 8 and 9.

Therefore, treatment decisions were made on account of SB results in 17.1% (6/35) of men undergoing radical treatment for prostate cancer, which was 5.77% of the whole cohort.

A review of the RP histology in the 3 men who went on to have RP showed that 2 had Gleason 3 + 4 csPCa, and 1 had a 1 cm lesion Gleason 3 + 3 csPCa. The RP specimens further provide evidence that without the SB, csPCa would not have been detected or treated in these patients.

In summary, these results are significant from a diagnostic and therapeutic standpoint as it shows that concomitant SB not only reduces false-negative biopsy results but also allows earlier diagnosis and reduces undertreatment in 1 in 20 men with prostate cancer.

## Discussion

The PROMIS trial has provided robust level 1b evidence that mpMRI is more sensitive than SB at detecting csPCa.^1^ Trans-rectal ultrasound-guided (TRUS) 12 core SB and MRI results were compared to template biopsy histology. The mpMRI sensitivity for csPCa mpMRI (defined as Gleason primary pattern 4 or any cancer ≥ 6 mm) was 93% compared to 48% for TRUS biopsy (*P* < .0001). With a negative predictive value of 72%-89%, this suggests 11%-28% of csPCa is missed on mpMRI.^1^ The international multicenter PRECISION Trial^2^ included 500 men with raised PSAs who were randomized to TRUS SB or pre-biopsy MRI. In the pre-biopsy MRI group, 72% of men had Likert 3-5 MRI results and went on to have a TB. In the group who had MRI TB, csPCa was found in 38% of men compared to the SB group, which detected csPCa in 26% (*P* = .005). Moreover, MRI TB detected fewer insignificant cancers in 9% of men compared to 22% of men in the SB group (*P* < .001). These results allowed clinicians to have confidence in avoiding biopsies in men where pre-biopsy MRI was normal (PI-RADS v2 score 1 + 2) and demonstrated the superiority of MRI-influenced TB over SB with respect to the csCDR. However, since the SB and TB biopsy trial arms were separate, the additive diagnostic value of each biopsy technique could not be assessed.

A Cochrane analysis, based on pooled data from 43 studies where template biopsy histology was compared to mpMRI findings, revealed that the sensitivity of biparametric MRI and mpMRI to detect ISUP ≥ 2 prostate cancer has a pooled sensitivity of 0.91 (95% CI: 0.83-0.95) and a pooled specificity of 0.37 (95% CI: 0.29-0.46).^8^ The pooled sensitivity and specificity for csPCa of SB was 0.63 (95% CI 0.19-0.93) and 1.00 (95% CI 0.91-1.00) when compared to template biopsies. The analysis reports that MRI will miss 9% of csPCa and that MRI TB will miss 20% of clinically significant cancer.^8^

Several prospective multicenter trials have examined the additional benefit of combined TB and SB: MRI First^3^ 4M Study,^23^ and sub-study results reported by Ahdoot et al.^18^ A total of 251 biopsy naive men with suspected organ-confined prostate cancer were included in the MRI-First trial.^3^ All men had mpMRIs followed by an SB with an operator blinded to the MRI. The TB was performed in those with PI-RADS 3-5 lesions. The csPCa was defined as ISUP ≥ 2. The csCDR was 37.5%. There was only a small and non-significant difference between the csCDR of TB 32.3% and SB 29.9% (detection ratio 1.08 *P* = .38). Furthermore, of those with csPCa, 14% were diagnosed with SB alone, 20% were diagnosed with TB alone, and 66% were diagnosed with both techniques, revealing the added, synergistic benefit of each^3^ type of biopsy. The 4M Study included 626 biopsy naïve patients with PSA ≥ 3 ng/mL.^23^ All patints in this study had pre-biopsy mpMRI and SB. Those patients with PI-RADS 3-5 lesions also had in bore MRI TB and SB. The csPCa was defined as ISUP ≥ 2. There was no significant difference in csCDR for TB at 25% compared to 23% for SB (detection ratio 1.09 *P* = .17), with TB showing much lower detection rates of insignificant cancer at 14% versus 25% for SB (*P* < .0001). An additional 7% of csPCa was detected on SB in those with PI-RADS 3-5 lesions.^23^

Pooled data from the Cochrane analysis show slightly higher rates of csPCa detection for TB than SB.^8^ The difference becomes more marked when examining higher ISUP grades and examining those with previous negative biopsies. This is evidenced by detection ratios for those with previous negative biopsies of 1.44 and 1.64 for ISUP ≥ 2 and ISUP ≥ 3 and only 1.05 and 1.09 in those who were biopsy naive. Specifically, results revealed that in the repeat-biopsy cohort, TB would miss approximately 10% of ISUP ≥ 2 Pca,^8^ comparable to our results. These sub-group analyses indicate the greater usefulness of MRI TB in the repeat biopsy setting and for accurately diagnosing higher-grade tumors. However, although TB may not outperform SB for the majority of patients on their first biopsy, it does allow greater confidence in getting an accurate diagnosis the first time, avoids repeat biopsies, and allows greater confidence in discharging a patient for PSA monitoring.

Our data support a growing field of evidence that although MRI TB is more sensitive than SB at detecting csPCa, SB adds to the number of patients diagnosed with csPCa in those already undergoing prostate biopsy. Our results showed TB has a higher CDR and csCDR and missed fewer csPCa than SB. The detection ratio of csCDR for TB compared to SB was 2.4 for any UCL 1 criteria and 2.7 for ISUP ≥ 2. These high detection ratios for TB over SB in this cohort are likely to result from the fact that it includes mainly previously biopsied patients, which are self-selected to have a low SB csCDR. Our data showed a much higher rate of insignificant cancer detected on SB and TB at between 26.9%-23.1% and 14.4%-30.8%, respectively (for <UCL1 criteria and ISUP 1 criteria). The percentage of csPCa missed on TB in our study is between 9.1% (2/22) (ISUP≥2) and 11.1% (4/36) (UCL 1 criteria). This evidence of additional csPCa diagnoses on concomitant SB is supported by results from the Cochrane analysis,^8^ 4M,^23^ MRI First study,^3^ and recently by Ahdoot et al.^18^ with clear and powerful results.

Ahdoot et al^18^ reported outcomes in 2103 men with PI-RADS 3-5 lesions on mpMRI who underwent Fusion MRI TB and SB. As high as 79% had undergone previous prostate biopsies. The 19.2% that underwent RP had a whole-mount histological examination of prostatectomy specimens examined. The study used ISUP ≥ 3 to define csPCa but also reported on ISUP ≥ 2.^18^ The CDR for TB was 51.5% and for SB was 52.5% with a combined CDR of 62.4%. Clinically significant cancer was found on biopsy in 43.7% when defined as ISUP ≥ 2 and in 22.2% when defined as ISUP ≥ 3. Additional ISUP ≥ 2 and ISUP ≥ 3 cancers were detected in all 2103 men by SB in 5.8% and 1.9%, respectively. Of those with ISUP ≥ 2 cancers and ISUP ≥ 3 cancers, this amounts to 13.4% and 8.8%, respectively.

Whole-mount RP histology was reviewed for pathological upgrading or downgrading in the 404 men who underwent RP. This reported that final histology was upgraded to ISUP ≥ 2 in 18.3% and to ISUP ≥ 3 in 8.7%, compared to the combined SB plus TB histology results, where 6.7% and 3.5% were upgraded, respectively,^18^ thereby indicating that SB plus TB provided significantly more accurate grading than TB alone.

This additional accuracy for prostate biopsy diagnosis in predicting true prostate pathology allows better patient counseling and reduces the risk of overtreating and undertreating prostate cancer due to the concern about inaccuracies in prostate biopsy.

The variation in the classification of csPCa and the importance for individual patient decisions not only lead us to have 2 definitions of csPCa in this study but also prompted us to assess the impact on clinical management, which showed 17.1% of men with csPCa and 5.77% (6/104) of men in this cohort had radical treatment offered to them based on SB results. Importantly, in our cohort, 2 of those 6 (with only 3 having RP histology to review) had ISUP grade group 2 RP histology, giving weight to the argument to proceed with concomitant SB.

The main limitation of this study is that the majority of patients in this cohort underwent biparametric MRI without gadolinium enhancement.

PI-RADsV2 uses T2-weighted imaging as the dominant sequence for transitional zone lesions and DWI using as the dominant sequence for peripheral zone lesions. Dynamic contrast enhancement (DCE) with gadolinium is of the most benefit in differentiating between PI-RADs 3 and PI-RADs 4 lesions.^22^ Furthermore, no significant difference in diagnostic test accuracy has been found on systematic review and meta-analysis comparing biparametric MRI and mpMRI in treatment-naive patients^23^; therefore, the lack of DCE is not anticipated to have significantly altered our results.

The fact that the clinician was not blinded to the MRI while taking SB could potentially have increased the SB CDR. However, our detection rates are in line with the SB CDR of previously published studies.

Accurate prostate biopsy is fundamental to enable clinicians to stratify risk and enable robust decision-making in patients with curable prostate cancer. Collectively, this study of mainly previously biopsied men has shown that 9.1%-11.1% of men would have a false negative or insignificant TB result. Moreover, 17.1% of patients with csPCa would not have proceeded to radical treatment if the SB had not been performed and RP specimens confirmed csPCa in all RP histology in this group. The superiority of TB over SB is evident in our data and our high detection ratios for TB compared to SB for csPCa which is consistent with meta-analysis examining results from a repeat biopsy setting. Our data support an emerging theme that concomitant SB in PI-RADS 3-5 patients provide an additional diagnosis of csPCa, and with this greater accuracy, greater confidence in biopsy results can be given. Consequently, the risk of overtreatment and undertreatment can be reduced.

## Figures and Tables

**Table 1.** Results

104 patients Underwent TB Using Fusion Technology Targeting PI-RADS 3-5 Lesions	Overall biopsy (n) = 104 UCL 1 Criteria Any Cancer > 6 mm or Primary Gleason 4	Overall biopsy (n) = 104 ISUP ≥ 2
Age (years), median (range)	68 (52-82)	
PSA (ng/mL), median (range)	9.3 (1.3-35.4)	
Prostate volume (mL), median (range)	60 (12.5-227)	
TB CDR, n (%)	47/104 (45.2%)	
SB CDR, n (%)	41/104 (39.4%)	
TB + SB, CDR n (%)	61/104 (58.7%)	
Clinically significant		
TB csCDR, n (%)	32/104 (30.8%)	19/104 (18.3%)
SB csCDR, n (%)	13/104 (12.5%)	7/104 (6.7%)
TB + SB csCDR, n (%)	36/104 (34.6%)	22/104 (21.1%)
Cancer missed on TB, n (%)	(61-47)/61 (23.0%)	
Cancer missed on SB, n (%)	(61-41)/61 (32.8%)	
Significant cancer missed on TB, n (%)	4/36 (11.1 %)	2/22 (9.1%)
Significant cancer missed on SB, n (%)	23/36 (63.9%)	14/22 (63.6%)
Clinically insignificant cancer detected on TB, n (%)	15/104 (14.4%)	32/104 (30.8%)
Clinically insignificant cancer detected on SB, n (%)	28/104 (26.9%)	24/104 (23.1%)
Clinically insignificant cancer detected on combined biopsy, n (%)	25/104 (24.0%)	39/104 (37.5%)

csCDR, clinically significant cancer detection rate; SB, systematic biopsy; TB, targeted biopsy.

**Table 2.** Influence of SB on Radical Treatment Decision in 6 Patients. Highlighted Boxes Indicate the Presence of csPCa. The Last Column Shows Radical Prostatectomy (RP) Histology/Treatment Option.

Patient Number	SB Left Histology	SB Right Histology	TB Histology	Treatment Decision Based on SB/TB	RP Histology/Radiotherapy/Active Surveillance
1	**1/5 cores Gleason 4 + 4 max 6 mm** **Significant both ISUP ≥ 2 and UCL1 criteria**	Negative	Right posterior ×6: negative Left posterior ×4: negative	Based on SB	RP: Multifocal T3a Gl 3 + 4 = 7
2	**1/6 cores** **Gle** **ason 3 + 3** **7 mm** **Significant UCL1 criteria**	**1/6 cores** **Gleason 4 + 3** **2 mm** **Significant both ISUP ≥ 2 and UCL1 criteria**	**Apex 4/5 cores Gleason 3 + 3** **Max 8 mm** **Significant UCL1 criteria**	SB upgraded to ISUP 3.	Radiotherapy
3	2/6 cores Gleason 3 + 3 Max 3 mm Not significant	**6/7 cores** **Gleason 3 + 3** **Max 6 mm** **S** **ignificant UCL1 criteria**	Left anterior 2/5 cores Gleason 3 + 3 2 mm + 1.5 mm Not significant	Based on SB	RP: T2c multifocal Gl 3 + 4 = 7
4	4/6 cores Gleason 3 + 3 Not stated NA	**3/6 cores** **Gleason 3 + 3** **Max 7 mm** **Significant UCL1 criteria**	Right peripheral 2/5 cores Gleason 3 + 3 1 mm + 1 mm Not significant	Based on SB	Patient offered radical treatment but chose to remain on AS.
5	5/6 cores Gleason 3 + 3 2 mm Not significant	4/6 cores Gleason 3 + 3 3 mm Not significant	Apex 2/4 cores Gleason 3 + 3 Max 3 mm Not significant	Based on both: Upstaging to T2c As left and right lobes positive	Radiotherapy
6	1/6 cores Gleason 3 + 3 Max 4 mm Not significant	Negative	Right anterior 2/3 cores Gleason 3 + 3 Max 4 mm Not significant	Based on both: Upstaging to T2c as left and right lobes positive	RP: right apex T2c Gl 3 + 3

AS, active surveillance; Gl, Gleason; RP, radical prostatectomy; SB, systematic biopsy; TB, targeted biopsy.
